# The complete mitochondrial genome of *Epinephelus Chlorostigma* (*Serranidae*; *Epinephelus*) with phylogenetic consideration of *Epinephelus*

**DOI:** 10.1080/23802359.2018.1436992

**Published:** 2018-02-10

**Authors:** Xun Du, Li Gong, Wei Chen, Liqin Liu, Zhenming Lü

**Affiliations:** National Engineering Laboratory of Marine Germplasm Resources Exploration and Utilization, College of Marine Science and Technology, Zhejiang Ocean University, Zhoushan, P. R. China

**Keywords:** *Epinephelus chlorostigma*, mitogenome, phylogenetic relationship

## Abstract

The complete mitochondrial genome of *Epinephelus chlorostigma* has been determined. It contains a typically conserved structure including 13 protein-coding genes, 22 tRNA genes, two rRNA genes and one control region, and the whole sequence was 16,894 bp in length. The overall base composition is A 28.70%, C 27.99%, G 16.28%, T 27.03%. Except *ND6* and eight tRNA genes, all other mitochondrial genes are encoded on the heavy strand. Phylogenetic tree was constructed based on 12 protein-coding genes sequences of 18 *Epinephelus* species, four species belong to Cephalopholis as outgroup, the result showed that *E. chlorostigma* is most closely related to *Epinephelus areolatus.* We sequenced the complete mitochondrial genome of *E. chlorostigma* to enrich the resource of molecular markers for examination of phylogenetic relationships in *Epinephelus*.

Groupers live in reefs or gravel in the temperate and tropical seas. There are over 100 species of grouper in the world, 90% of which are in Asian sea areas. The Brown-spotted grouper, *Epinephelus chlorostigma,* is one of these valuable varieties which distributed in the Indian Pacific Ocean, it belongs to family Serranidae, genus *Epinephelus*. In order to find new DNA markers for the future research of population genetics and phylogenetics and taxology, we determined the complete mitogenome of *E. chlorostigma* (GenBank accession number no. MG739436) by PCR amplification and primer walking sequence method, it would be useful for further understanding the evolution of ratite and conservation genetics of *Epinephelus*.

In this study, we reported the complete mitogenome of *E. chlorostigma* from the South China Sea (18°3′35″N 109°24′19″E). Samples stored in a refrigerator of −80 °C with accession number 20171017EC01. The complete mitochondrial genome of *E. chlorostigma*, with 16,894 bp in length, includes 13 protein-coding genes, 2 ribosomal RNA (rRNA) genes, 22 transfer RNA (tRNA) genes and 1 control region (D-Loop). Genes encoding on the genome are similar among all Perciformes (Liu et al. 2016; Ayala et al. 2017; Kim et al. 2017), with the exception of *ND6* and eight tRNA genes (Gln, Ala, Asn, Cys, Tyr, Ser, Glu, Pro), all other mitochondrial genes are encoded on the H-strand (Liu et al. 2016). The total length of the 13 protein-coding genes was 11,427 bp, which corresponded to 67.64% of the whole mitochondrial genome. The overall base composition is A 28.70%, C 27.99%, G 16.28%, T 27.03%. The A + T content (56.69%) is higher than G + C content (43.31%), in common with other Perciformes mitogenomes (Bian et al. 2016; Chen et al. 2016; Ayala et al. 2017; Shi et al. 2018). Eleven protein-coding genes start with ATG except *COX1* with GTG and *ATP6* with ATA. For the stop codon, eight protein-coding genes stop with TAA except *ND5* with TAG, *COX2, ND3, ND4* and *CYTB* with an incomplete T. The 22 tRNA genes vary from 69 to 76 bp in length. The 12S rRNA is located between tRNA-Phe and tRNA-Val genes is 953 bp in length (Kim et al. 2017). The 16S rRNA is located between tRNA-Val and tRNA-Leu genes (Song et al. 2016) and is 986 bp in length. The OL (origin of L-strand replication) is located between tRNA-Asn and tRNA-Cys, the same with other Perciformes (Guo et al. 2016), and is 43 bp in length. The control region was 1090 bp in length, localized between tRNA-Pro (TGG) and tRNA-Phe (GAA) genes.

Sequence alignment was conducted by BioEdit (Hall 1999). And phylogenetic tree was constructed using maximum-likelihood (ML) method based on the 12 protein-coding genes (except *ND6* gene) of 18 available representative species in *Epinephelus* and four species belong to Cephalopholis as an outgroup (Figure 1). The result shows that *E. chlorostigma* is most closely related to *Epinephelus areolatus*. We expect the present results will provide an important data set for phylogenetic and taxonomic analyses of genus *Epinephelus* species.

**Figure 1.**
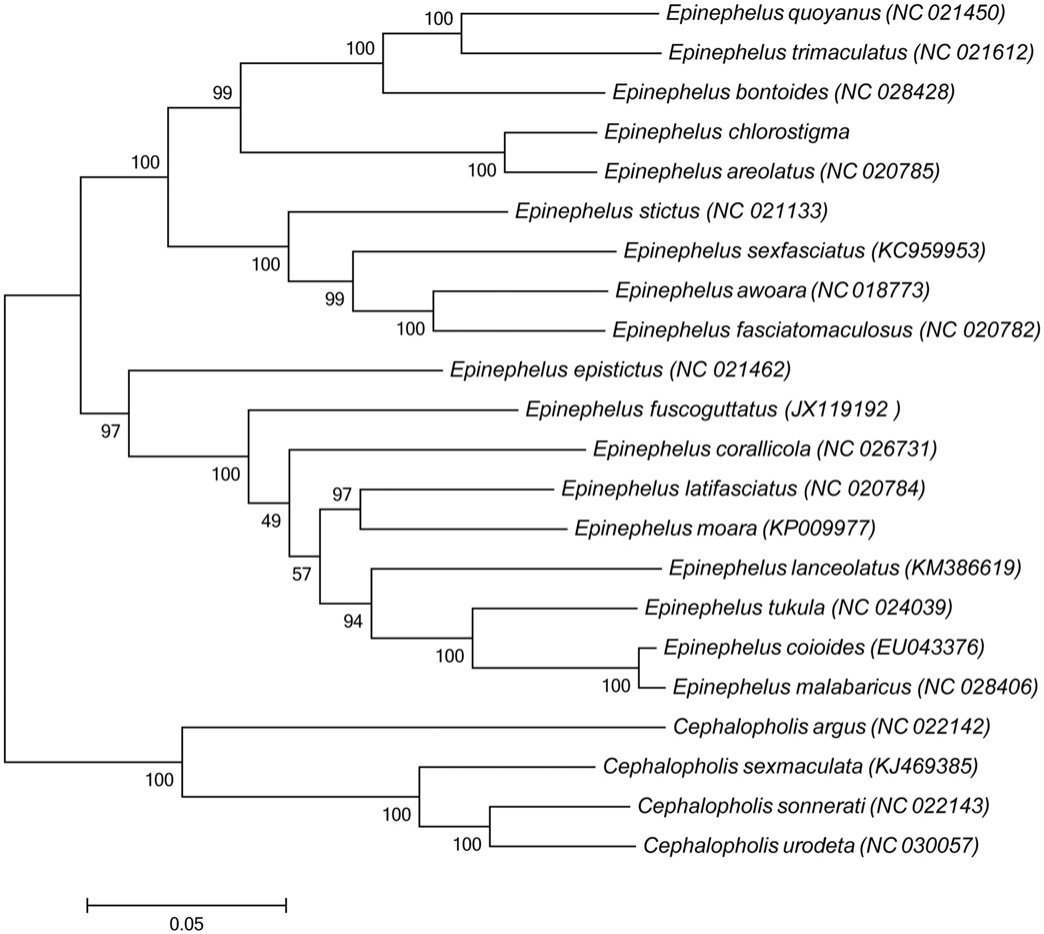
Phylogenetic tree was constructed based on 12 protein-coding genes (except ND6 gene) of 18 *Epinephelus* complete mitogenome. The black dot indicated the species in this study. The number at each node is the bootstrap probability. The number before the species name is the GenBank accession number.
